# First case of detection of *Plasmodium knowlesi *in Spain by Real Time PCR in a traveller from Southeast Asia

**DOI:** 10.1186/1475-2875-9-219

**Published:** 2010-07-27

**Authors:** Thuy-Huong Ta Tang, Ana Salas, Marwa Ali-Tammam, María del Carmen Martínez, Marta Lanza, Eduardo Arroyo, Jose Miguel Rubio

**Affiliations:** 1 Malaria & Emerging Parasitic Diseases Laboratory, Parasitology Department, National Centre of Microbiology. Instituto de Salud Carlos III, Cra. Majadahonda Pozuelo Km. 2, Majadahonda, 28220 Madrid, Spain; 2 Infectious Diseases Department. Hospital Universitario de La Princesa, Madrid, Spain; 3 Genetic Forensic and Population Genetic Laboratory, Toxicology and Sanitary Legislation Department, Faculty of Medicine, Universidad Complutense de Madrid, Spain

## Abstract

Previously, *Plasmodium knowlesi *was not considered as a species of *Plasmodium *that could cause malaria in human beings, as it is parasite of long-tailed (*Macaca fascicularis*) and pig-tailed (*Macaca nemestrina*) macaques found in Southeast Asia. A case of infection by *P. knowlesi *is described in a Spanish traveller, who came back to Spain with daily fever after his last overseas travel, which was a six-month holiday in forested areas of Southeast Asia between 2008 and 2009. His *P. knowlesi *infection was detected by multiplex Real time quantitative PCR and confirmed by sequencing the amplified fragment. Using nested multiplex malaria PCR (reference method in Spain) and a rapid diagnostic test, the *P. knowlesi *infection was negative. This patient was discharged and asymptomatic when the positive result to *P. knowlesi *was reported. Prior to this case, there have been two more reports of European travellers with malaria caused by *P. knowlesi*, a Finnish man who travelled to Peninsular Malaysia during four weeks in March 2007, and a Swedish man who did a short visit to Malaysian Borneo in October 2006. Taken together with this report of *P. knowlesi *infection in a Spanish traveller returning from Southeast Asia, this is the third case of *P. knowlesi *infection in Europe, indicating that this simian parasite can infect visitors to endemic areas in Southeast Asia. This last European case is quite surprising, given that it is an untreated-symptomatic *P. knowlesi *in human, in contrast to what is currently known about *P. knowlesi *infection. Most previous reports of human *P. knowlesi *malaria infections were in adults, often with symptoms and relatively high parasite densities, up to the recent report in Ninh Thuan province, located in the southern part of central Vietnam, inhabited mainly by the Ra-glai ethnic minority, in which all *P. knowlesi *infections were asymptomatic, co-infected with *P. malariae*, with low parasite densities and two of the three identified cases were very young children under five years old.

## Background

Recent reports from Asia suggest the possibility that *Plasmodium knowlesi*, is emerging as an important zoonotic human pathogen [1]. The natural hosts of *P. knowlesi *are the long-tailed (*Macaca fascicularis*) and pig-tailed (*Macaca nemestrina*) macaques [2], commonly found in Southeast Asia. *Plasmodium knowlesi *has a quotidian (24 h) asexual blood cycle, the shortest among primate malarias and produces daily fever peaks in its hosts, causing severe malaria if not treated [3]. At present, *P. knowlesi *transmission is restricted to the *Anopheles leucosphyrus *group of mosquitoes, which currently comprises 20 species [4]. The range of the *A. leucosphyrus *group overlaps with the long-tailed and pig-tailed macaques, and naturally acquired within this range. *Plasmodium knowlesi *was first described in 1931; in 1932 was experimentally shown to be infectious to humans [3]. The first natural infection in humans was reported in 1965 [5] in a man from the United States after a visit to Peninsular Malaysia. In 1971, there was a presumptive case in a man of Malaysia [6]. No other reports were published on naturally acquired *P. knowlesi *infections in humans until 2004 [3]. *Plasmodium knowlesi *infections in human are not exclusive to Malaysia, but can also appear in China [7], in Thailand [8], Philippines [9], Singapore [10] and Indonesian Borneo [11]. *Plasmodium knowlesi *can be misidentified on a blood smear as the morphology of the blood-stage forms share similarities with *Plasmodium malariae *and *Plasmodium falciparum*, such as "bands forms" attributed to *P. malariae*, and delicate ring forms as seen with *P. falciparum *infections [2,12]. Currently, PCR assay and molecular characterization are the most reliable methods for detecting and diagnosing *P. knowlesi *infection [13-15]. Rapid diagnostic tests kits may or may not recognize *P. knowlesi *because of their specificity [16,17]. The disease can be treated using already existing anti-malarial therapy such as mefloquine and chloroquine. This report describes the first case in Spain and the third in Europe of naturally acquired infection with *P. knowlesi *in a Spanish traveller while staying in Southeast Asia. Diagnosis was confirmed by multiplex Real time quantitative PCR and subsequent sequencing of the real-time PCR fragment.

## Case presentation

In March 2009, a 39-year-old Spanish man presented to a hospital in Madrid (Spain) with a fifteen-day history of daily and evening fever spikes, temperature of up to 40°C, artralgia, myalgia, low back pain, chills and malaise. The patient had recently returned from a six-month holiday in cities of Southeast Asia between 2008 and 2009: Bangkok (Thailand), Banda Aceh and Pulau Weh (Indonesia) for three months, Kuala Lumpur (Malaysia) and Hanoi (North of Vietnam), places where human malaria is endemic. He reported being in contact with simians and staying in rural areas. He did not remember being bitten by mosquitoes, however some of his fellow travellers suffered Dengue and malaria attacks. He began malaria prophylaxis with mefloquine but due to adverse effects he changed to Malarone^®^. He reported to have taken 80% prophylaxis. In view of the complaints of the patient, the physician decided to admit him on 31^st ^March 2009. Physical examination showed a remarkable hepatomegaly and splenomegaly.

Laboratory investigations showed no anaemia (haemoglobin 12.7 g/dl, reference range 12-17), with normal erythrocyte count (4,46 × 10^6 ^/mm^3^, reference range 3,5-5,5), moderate leucopaenia (3.82 × 10^3 ^/mm^3^, reference range 4-10), thrombocytopaenia (platelet count 86 × 10^3 ^/mm^3 ^, reference range 115-450), and some liver function abnormalities (serum alanine aminotransferase 93 U/L, reference range 5-41; aspartate aminotransferase 43 U/L, reference range 4-38; gamma-glutamyltransferase 78 U/L, reference range 11-49; alkaline phosphatase 71 U/L, reference range 40-129; and total bilirubin 1.1 mg/dl, reference range 0.2-1.3).

Urine analysis, blood sugar, blood urea nitrogen, and creatinine were normal. Renal function was normal. Patient's peripheral blood samples were forwarded to the Diagnostic Reference Laboratory in Madrid to be tested to a possible bacterial, viral or parasitic infection. To control the patient's fever physicians gave intravenous paracetamol and hydration. Fever was of 38.5°C during the first week, restoring to normal in the second week temperature and analytic parameters. It was not necessary any kind of antibiotic to manage the clinical state of the patient.

Dengue, Q fever, rickettsiosis were ruled out with negative serology and reverse transcription-polymerase chain reaction. Likewise, one sample was sent to the Malaria & Emerging Parasitic Diseases Laboratory (2^nd ^April 2009). The patient DNA was extracted by automated extraction on the *QIAcube*™. *QIAamp^® ^DNA Mini and Blood Mini Kit. Qiagen*. The result obtained with nested multiplex malaria PCR (polymerase chain reaction) [18] was negative for the four human *Plasmodium *species. This nested PCR only can detect and differentiates between the four human *Plasmodium *species.

Meanwhile, this laboratory was evaluating a DNA-based diagnostic method by real time PCR targeting the small subunit ribosomal RNA (SSU rRNA) genes of all *Plasmodium *species (human and non-human). The patient's sample was positive for malaria DNA. The real-time PCR generated a PCR product which was positive for specific-*Plasmodium *probe. This assay include one set of genus-specific primers and one set of fluorescent energy transfer hybridization probes: a genus *Plasmodium*-specific probe, a *P. falciparum *species-specific probe and a third probe specific for *P. vivax*. The genus *Plasmodium*-specific probe hybridizes to amplicons from all four human and non-human *Plasmodium *species. Based on melting curve analysis, all *Plasmodium *are indistinguishable among them. In this real time PCR method positive and negative isolates are always included as positive and negative controls, respectively. The parasitaemia level calculated by the multiplex real time quantitative PCR on the basis of a standard curve performed on the ten-fold dilutions of infected blood sample (parasitaemia proportion was identified by microscopy and expressed as number of parasites per white blood cell in a thin blood film by examining over 100 fields and multiplied by 8,000 and as a percentage of erythrocytes) within uninfected erythrocytes from healthy individuals with known baseline erythrocyte counts, was approximately of 250 parasites/μl blood or equivalent to parasitaemia 0.003%.

**Figure 1:**
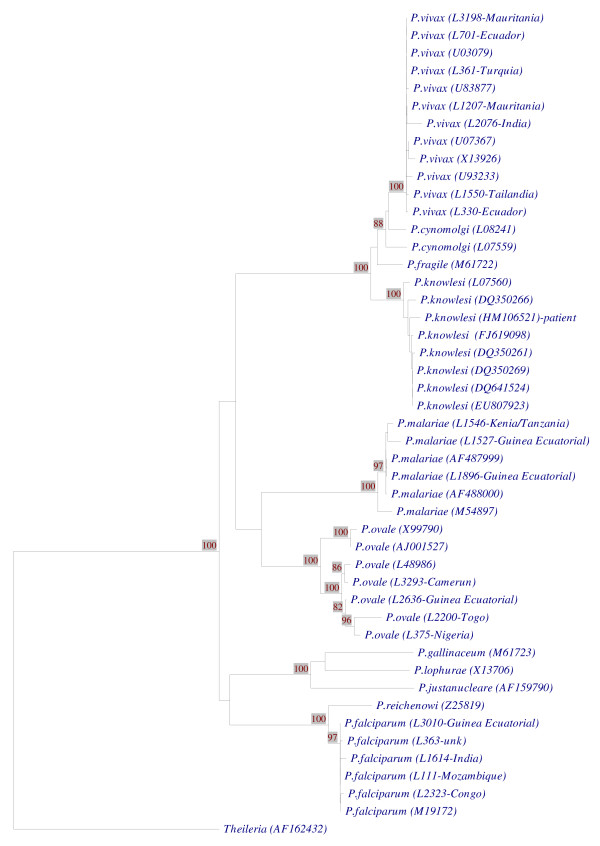
**Phylogenetic tree comparing our Spanish case (GenBank accession number **HM106521**) with other *Plasmodium *species identified in our laboratory as well, but not submitted to the GenBank (identified as "L plus a internal number" given in our Malaria Laboratory followed of the country name in the cases where this is known) and with known *Plasmodium *A-type SSU rRNA sequences from GenBank (accession numbers are indicated in parenthesis)**. The sequence of our patient clusters with all other *P. knowlesi *strains.

The amplification product generated by multiplex real time quantitative PCR was sequenced and this sequence was compared with known *Plasmodium *SSU rRNA sequences and the sequence of the clinical isolate strongly coincided with *P. knowlesi *SSU rRNA sequences transcribed during asexual stages (Figure 1). This sequence of 812 bp has been submitted to the GenBank data base with the following accession number HM106521.

**Figure 2:**
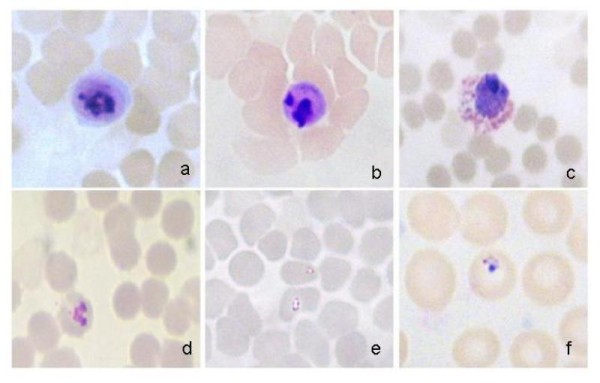
**Giemsa-stained thin blood films of the Spanish traveller infected with multiplex Real Time quantitative PCR-confirmed *Plasmodium knowlesi***. By microscopy, inside the infected erythrocytes, structures compatible with *Plasmodium *can be appreciated. a-b: mature trophozoites; c: gametocyte; d: indefinite stage; e-f: early trophozoites.

After this amazing finding in this sample, a thorough analysis was performed on it. In a rapid diagnostic test for malaria (Binax Now Malaria Test; Binax, Inc., USA), the sample was both negative for *P. falciparum *histidine-rich protein 2 and for pan-malarial aldolase antigen, suggesting a non-*Plasmodium *infection. Retrospective examination of Giemsa-stained thin blood films showed infected erythrocytes with an inconclusive morphologic appearance. The parasite structure found inside the erythrocytes was compatible with *Plasmodium *which suggested a possible infection by *Plasmodium*, in agreement with multiplex Real time quantitative PCR results (Figure 2). The serodiagnosis of malaria caused by *P. falciparum *was negative (Falciparum-Spot IF, Biomérieux S.A., France).

The patient was already discharged since 13^th ^April 2009 when this positive result to *P. knowlesi *was reported by early May (6^th ^May 2009). The patient was contacted in June (due to this patient is very fond of travelling, his whereabouts was hard to determine) for a new consultation and treated with chloroquine despite being asymptomatic. A month later, in the follow-up visit he continued being well. Previous to the treatment, a blood sample was taken again and this time was only sent to the Malaria & Emerging Parasitic Diseases Laboratory (16^th ^June 2009). The second blood sample was both negative by microscopy and by real time PCR (results given 24^th ^June 2009). This shows that there was a conflicting result between the first and the second blood sample. In order to rule out any possible error in the handling and/or identification of the samples belonging to this patient, an Individual Genome Mapping was done by Genetic Forensic and Population Genetic Laboratory. Both samples were typed through AmpFlSTR^® ^Identifiler^® ^PCR Amplification Kit (Applied Biosystems, USA) for 15 authosomal STRs ("short tandem repeats"), widely used in the forensic and identification field. Protocol was performed according to manufacturer instructions. The analysis of the fragments was performed in ABI PRISM^® ^3730 Genetic Analyzer. Both samples yielded identical STRs profiles, and were consequently considered from the same individual origin.

The large focus of human infection with *P. knowlesi *has been reported in Sarawak, Malaysia Borneo and later in several countries of Southeast Asia [7-11], as well as in US, Finnish and Sweden travellers [1,19,20] and together with this present report, this indicates that humans are indeed susceptible to infection by simian parasites. Travellers can spread new and re-emerging infectious diseases that initially appear in developing countries, and they act as ideal sentinels for the early detection of these diseases [21]. This is the first report of human *P. knowlesi *infection in Spain, with very unusual characteristics, given that most previously reported cases were symptomatic (between mild and severe infection) and treated with anti-malarial drugs. Even though there are very few asymptomatic cases of *P. knowlesi *human infection [22], this monkey malaria may be less severe among humans than was previously thought. This does not discard that specific PCR primers for *P. knowlesi *should be included to provide valuable diagnostic information [3], and laboratory clinicians and physicians should become more aware of this disease. Recently published reports indicate that the primers described by Singh *et al *[3] have the possibility of cross-hybridization with *P. vivax *[22,23].

The reason why our patient did not receive any anti-malarial treatment even though he had some symptoms compatible with malaria disease such as daily fever, thrombocytopaenia, hepatomegaly and splenomegaly, and he was clinically well in the second consultation without anti-malarial therapy cannot be explained. There is a possibility that residual prophylactic drug levels may have helped control the parasitaemia and severity of symptoms in this patient before hospital admission. A cross-contamination was dismissed from the beginning because in Malaria & Emerging Parasitic Diseases Laboratory had never worked before with *P. knowlesi *DNA and this case showed an unexpected parasite species.

## Conclusion

*Plasmodium knowlesi *infection is normally considered as a parasite of macaques, humans who work at the forest fringe or enter the rainforest to work are at risk of infection [24]. Clearly, blood-smear diagnosis alone is inadequate to confirm whether a patient has *P. malariae *or *P. knowlesi *infection. Clinical and Diagnostic Reference Laboratories should now include the capacity to detect *P. knowlesi *in their validated PCR-based tests for malaria, as current laboratory methods for species differentiation target only the four human plasmodia species [25]. Clinics in the West should be aware of *P. knowlesi *as a possible cause of human malaria. The prevalence of naturally acquired primate malaria in human may be underestimated [26].

The multiplex Real time quantitative PCR and subsequent sequencing the PCR fragment confirmed that the patient had *P. knowlesi *parasite. Apparently the patient had the infection by *P. knowlesi *but he developed an uncomplicated knowlesi malaria disease and he improved without receiving treatment for malaria. It might the prophylaxis with Malarone^® ^was effective against erythrocytic stage, but that does not avoid the infection. Most of the parasite forms seen on the blood film were gametocytic stage (this form does not produce symptoms in human and it can remain more time in blood). This case is uncommon, but is not the only, as there are three more atypical human *P. knowlesi *infections reported from central Vietnam [22]. Low parasitaemia in this patient (250 parasites/μl blood or equivalent to parasitaemia 0.003%) may have caused the lack of reactivity with the pan-malarial aldolase antigen; low parasitaemia will not be detected although a negative test result does not exclude a *P. knowlesi *infection [17]. However, parasites have been identified by microscopy in spite of low parasitaemia because a positive result was expected and many hours were spent to detect it [27].

Although very little is known about *P. knowlesi *infection, for the time being, out of Southeast Asia, it would be suitable or advisable to design specific *P. knowlesi *primers or to include them in the current validated PCR-based tests from Reference Laboratories.

## Consent

Oral informed consent was obtained from the patient for publication of this case report and any accompanying images after explanation of the report objectives.

## Competing interests

The authors declare that they have no competing interests.

## Authors' contributions

THT, JMR, AS and EA wrote the paper, AS and MDCM were the physicians responsible for the patient, JMR supervised molecular characterization of parasite, conceived the study, its design and coordination, analyzed the phylogenetic tree of SSU rRNA sequences and corrected the manuscript, THT carried out the multiplex real time quantitative PCR, analysed the sequence alignment and drafted the manuscript, EA was responsible for Individual Genome Mapping, ML performed the patient DNA isolation, archive blood sample, stained and examined the slides by microscopy, MAT participated in the sequence alignment. All authors have read and approved the final manuscript.
